# Metro-Peritonitis, — with Abdominal Abscess Successfully Opened in the Linea Alba

**Published:** 1873-10

**Authors:** A. R. Kilpatrick

**Affiliations:** Navasota, Texas


### METRO-PERITONITIS, — WITH ABDOMINAL ABSCESS SUCCESSFULLY OPENED IN THE LINEA ALBA.

BY A. R. KILPATRICK, M. D., NAVASOTA, TEXAS.

Some years ago I was called to attend a young unmarried woman who lived in the country, who while the catamenia were on her, got her feet wet, which caused sudden suppression, with high fever and other alarming derangements of the system. A physician living near by was called to attend her, who seemed not to comprehend the case, and so far forgot his professional responsibilities as to say to the family that she was pregnant. This so incensed her friends that the doctor was summarily dismissed, without intimating to her the cause. I was then called in, although distant fifteen miles.

I found her delirious, face flushed, eyes injected, pulse high, full and rapid, with great tenderness and tumefaction of the abdomen, especially over the uterus. In fact, there was a clear case of metro-peritonitis, requiring prompt and energetic treatment.

I bled her freely from the arm, which moderated the arterial excitement, and restored her to consciousness in a short time. Applied scarified cups as much as could be borne, and followed these with a large blister over the entire abdomen. Gave calomel and opium, and followed with oil and turpentine, which acted well. Dressed the blister with large soft poultices, and continued the calomel in alterative doses. She had been sick over three days when I first saw her. On my second visit there was effusion in the peritoneum, and I apprehended she would sink from so extensive and serious suppuration, as well as pyemic poisoning.

Feeling assured that death must ensue unless the effused liquid could be removed, I determined to open into the sac gradually, as at that day and time it was deemed highly dangerous to introduce a trochaz, or injure the peritoneum. I determined to adopt the plan which is followed in opening hepatic and psoas abscesses. Finding the greatest accumulation and bogginess on the mesial line between the umbilicus and pubis, I cut down with a bistoury midway between these points immediately on the linea alba, through the integument, the fascia and tendon, not quite through to the peritoneal lining, and introduced lint down to the bottom of the incision, retaining it with adhesive strips.

After forty-eight hours, I removed the lint, and about six ounces of pus gushed out to the great relief of the patient. By changing her position and using pressure, a considerable quantity was removed.

Tonics were used and nutritious diet allowed — poultices continued, and the abdomen bandaged as tightly as she could bear. No injections were made into the sac, but she went on to a complete cure, and the orifice healed in due time. Her health after that was as good as before the illness.

This case occurred twenty-seven years ago, when it was considered dangerous to introduce a trochaz into the peritoneal sac, and only done in extreme cases, or in ascites. I am now satisfied that there is far too much timidity felt by practitioners about tapping this and the plural sac. Numbers of persons are allowed to die who might be saved by drawing off serum and sero-purulent accumulations.

Dr. H. R. Storer, of Boston, says there is no more danger in tapping thoracic empyema than a common abscess, or a scrotal hydrocele. It requires time, and a long time, for the profession to divest itself of the dogmas of antiquity.

